# A Rare Case of Posterior Fossa Tumor and Central Precocious Puberty: Case Presentation and Review of the Literature

**DOI:** 10.3390/neurolint13040053

**Published:** 2021-10-20

**Authors:** Roberta Rana, Teresa Perrillo, Nicola Santoro, Federica Ortolani, Raffaella Messina, Mariachiara Resta, Ilenia Perrucci, Giuseppe Ingravallo, Gerardo Cazzato, Massimo Grassi, Sabino Pesce, Francesco Signorelli

**Affiliations:** 1 Department of Pediatric Oncology and Hematology, University “Aldo Moro” of Bari, 70124 Bari, Italy; roberta.rana90@gmail.com (R.R.); terryperillo@hotmail.com (T.P.); nico.santoro1956@libero.it (N.S.); grassimassimo@hotmail.it (M.G.); 2 Department of Metabolic Diseases, Clinical Genetics and Diabetology, Giovanni XXIII Children’s Hospital, 70124 Bari, Italy; federicaortolani@hotmail.com (F.O.); pscsbn@libero.it (S.P.); 3 Division of Neurosurgery, Department of Basic Medical Sciences, Neurosciences and Sense Organs, University “Aldo Moro” of Bari, 70124 Bari, Italy or signorelli2007@gmail.com; 4 Division of Neuroradiology, Department of Basic Medical Sciences, Neurosciences and Sense Organs, University “Aldo Moro” of Bari, 70124 Bari, Italy; mariachiararesta@yahoo.it (M.R.); ilenia.perrucci@policlinico.ba.it (I.P.); 5 Department of Emergency and Organ Transplantation—Section of Pathology, University “Aldo Moro” of Bari, 70124 Bari, Italy; giuseppe.ingravallo@uniba.it

**Keywords:** CPP, ganglioglioma, SNC, neuroradiology

## Abstract

Central precocious puberty (CPP) is a condition that causes early gonadotropin-dependent sexual development; CPP is idiopathic in girls in most cases, whereas more than 50% of boys have an identifiable etiology. We conducted a qualitative systematic review following the ENTREQ (enhancing transparency in reporting the synthesis of qualitative research) framework. A search was made in MEDLINE/Pubmed and MeSH Database using the terms “precocious puberty” AND “brain tumor” OR “posterior fossa tumor” OR “cerebellar tumor” OR “infratentorial tumor”, identifying five cases of pediatric patients with infratentorial tumors and CPP and a case of cerebellar ganglioglioma without hypothalamic−pituitary−gonadal axis involvement and/or intracranial hypertension. Our work highlights the importance of a multidisciplinary approach and extensive central nervous system imaging for patients presenting with CPP in order to detect possible tumor association. Moreover, we believe that this manuscript could contribute to stimulate other research because the exact mechanism of CPP in infratentorial brain lesions has not been understood yet.

## 1. Introduction

Precocious puberty (PP) is defined as the development of secondary sexual characteristics before the age of eight years in girls and nine years in boys [1,2]. PP may be classified as gonadotropin-dependent, namely true or central puberty (CPP), and gonadotropin independent, i.e., pseudo-PP or peripheral PP [1]. The prevalence of precocious puberty has been estimated to be 10–20-fold higher in girls compared with boys [3]. CPP in girls is idiopathic in most cases, whereas more than 50% of boys with CPP have an identifiable etiology [4]. Central nervous system tumors, infections, congenital defects, ischemia and radiation injury can cause secondary CPP [1,3]. Therefore, brain magnetic resonance imaging (MRI) must be performed in all children with PP, mostly in boys [5,6,7]. Among brain tumors, low-grade gliomas, pineal tumors, craniopharyngiomas, hypothalamic hamartomas may cause CPP because of the direct disruption of normal prepubertal inhibition of the hypothalamic-pituitary-gonadal axis [3]. Rare cases of posterior fossa tumors presenting with CPP have been described [8,9,10,11,12]. Here we report the case of a young boy with CPP and posterior fossa ganglioglioma (GG).

## 2. Case Presentation (Material)

**Figure 1:**
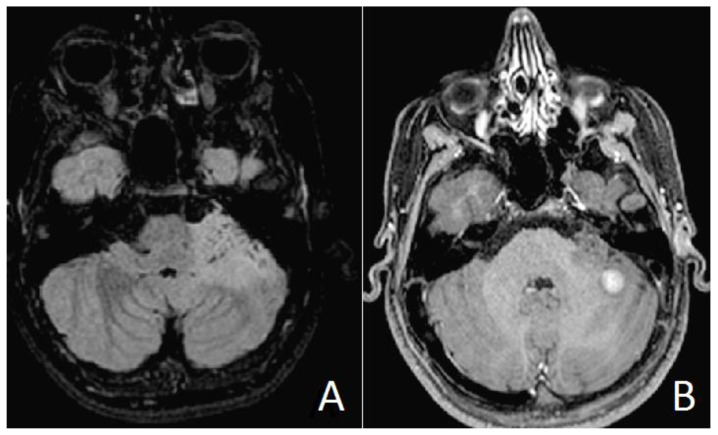
Preoperative brain MR images. Axial FLAIR T2 weighted images (**A**) showing microcystic areas inside the left hemispheric cerebellar lesion, infiltrating the middle cerebellar peduncle. Axial gadolinium-enhanced T1 weighted images (**B**) exhibiting a solid central enhanced nodule.

An 11-year-old boy was referred to our pediatric endocrinology department for suspect PP (onset of pubic hair at the age of 8.5). The patient’s weight was 49 kg (75–90p), height was 156.3 cm (97p) (Tanner’s target height 165.6–176.6 cm) and testicular volumes were 15 mL (Tanner stage 4 puberty). The bone age was three years older than the chronological age (14 years old according to Greulich Pyle Atlas). Laboratory work up revealed basal LH 2.94 mUI/mL (normal range < 0.3) FSH 3.58 mUI/mL (normal range 0.51–2.82), testosterone 4.23 ng/mL (normal range 0.026–0.055), free testosterone 23.15 pg/mL (normal range 0.82–15.4). After LH-FSH-releasing hormone test, a peak LH value of 35.1 mUI/mL and a peak FSH value of 9.01 mUI/mL (after 4 h) were detected. ACTH stimulation test was performed: 17 OH-P rose from a basal value 1.56 ng/mL to 6.25 ng/mL at 60 min; levels of DHEA-S, delta-4 androstenedione and cortisol remained within normal range. Late onset (non-classic) 21-hydroxylase deficiency was excluded by genetic analysis. Plasma thyroxin, thyroid-stimulating hormone, serum beta-hCG and alfa-fetoprotein concentrations were within normal range for age. One month after pediatric endocrinology consultation, brain MRI detected a mixed solid and microcystic left hemispheric cerebellar lesion growing into the cerebellomedullary and cerebellopontine cisterns, partly infiltrating the middle cerebellar peduncle, with a small extension into the jugular foramen. The solid component showed an inhomogeneous hyperintensity on T2-weighted turbo spin echo (TSE) and fluid attenuated inversion recovery (FLAIR) sequences. T1-weighted contrast-enhanced sequences showed two foci of centimetric nodular enhancement in the posterior lobe. Hydrocephalus was absent. Sagittal post-contrast T1-weighted images of the spine showed no leptomeningeal spread (Figure 1).

The patient was diagnosed with CPP and started gonadotropin releasing hormone (GnRH) agonist treatment (triptorelin acetate 3.75 mg every 90 days). He was referred to the pediatric oncology department for preoperative evaluation.

The patient did not complain of neurological symptoms and neurological examination was unremarkable.

**Figure 2:**
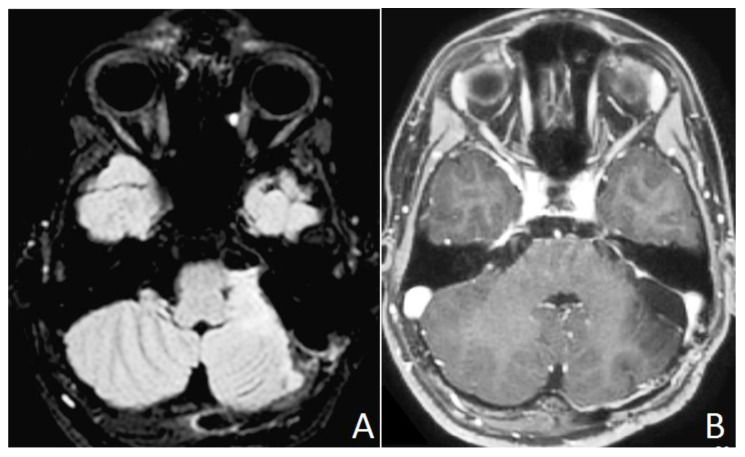
Postoperative brain MR images. A residual infiltration in the upper portion of the middle cerebellar peduncle is evident in axial FLAIR T2 weighted images (**A**). No residual contrast enhancement is evident after gadolinium administration in T1 weighted images (**B**).

Chest x-ray, abdomen and testicular ultrasound, cardiological, neuropsychiatric and genetic consultation did not reveal any abnormality. A minimal left hypoacusia was detected by otolaryngologists. The patient was operated on via a left retrosigmoid approach on lateral position. Intraoperative neuromonitoring (IONM) was performed recording brain stem auditory evoked potentials (BAEPs), motor and somatosensory evoked potentials and V VII, IX, X, XI cranial nerve monitoring. Baseline BAEPs showed a decreased function on the left. During and after surgery no further changes were recorded. Early postoperative MRI showed a small tumor residue in the middle cerebellar peduncle, hyperintense on T2-weighted TSE and FLAIR sequences, with no contrast enhancement on T1-weighted images (Figure 2).

Postoperative neurological examination reported a mild sensorineural left hearing loss and a transient diplopia. The boy was discharged on postoperative day 8.

**Figure 3:**
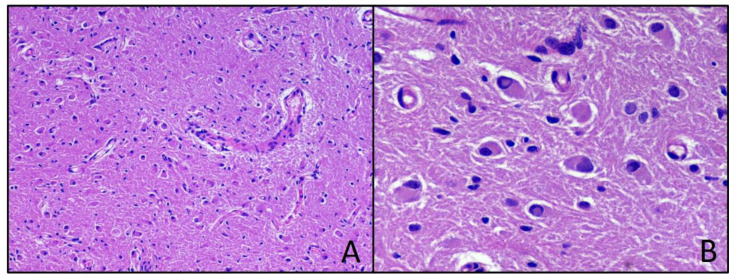
Ganglioglioma. Hematoxylin-eosin-stained photomicrographs with original magnification 100× (**A**) and 400× (**B**).

Histopathological features were consistent with a combination of aggregates and nests of neuronal elements, that were comprised in a background of neuropil containing benign glial elements, set in a reticulin containing stroma. Ganglion cells were large with multiple processes, nuclei were large, rounded and appeared somewhat hyperchromatic, with large nucleoli, without binucleate forms, cytomegaly, pleomorphism, mitoses and necrosis. Tumor margins were well-circumscribed, without leptomeningeal infiltration. Immunostaining for glial-fibrillar acid protein and chromogranin A were strongly positive in ganglion cells, but not in neuronal cells, which were strongly positive for synaptophysin. Ki67/MIB-1 labelling involved only glial component and was 1.0% (Figure 3). Final diagnosis was GG, grade I WHO 2016 [13].

Progression of puberty was clinically restrained, testicular volume decreased from 15 mL to 10 mL and GnRH treatment was maintained, with the indication to further evaluate biochemical response. Diplopia improved, but the auditory brainstem response confirmed left central auditory tract dysfunction. Brain MRI revealed stable findings with no evidence of tumor relapse.

### 2.1. Methods

We conducted a qualitative systematic review following the ENTREQ [14] (enhancing transparency in reporting the synthesis of qualitative research) framework. This methodology was chosen as it better describes qualitative research according to the guidelines for reporting systemic reviews at: http://www.cochrane.de/de/LeitlinienForschungsberichte (accessed on 4 May 2021). A case-based literature search of pediatric cases of CPP associated with posterior fossa tumor was performed. The main search was conducted through PubMed and MeSH Database and the terms used were “precocious puberty” AND “brain tumor” OR “posterior fossa tumor” OR “cerebellar tumor” OR “infratentorial tumor”. Only full text papers (case series, reviews and research studies) published between 2001 and 2021 in peer-reviewed journals were included in the study. A systematic approach to the collected data and a collegial discussion between the authors led to a final version of the manuscript.

### 2.2. Results

We identified a total of 191 records through database searching according to our selection criteria. All publications were obtained through MEDLINE/PubMed. Twenty-four studies were duplicate and were excluded from our review. A total of 167 peer-reviewed papers were screened based on this review’s focus (CPP in pediatric patients with posterior fossa tumor). Five papers describing cases of pediatric patients with infratentorial tumors and CPP were included for the qualitative analysis; 162 were found not relevant and were excluded (Table S1).

Four out of five tumors were in the cerebellum, two medulloblastomas [8,11] and two pilocytic astrocytomas [9,12]. All those patients received surgery as the main tumor treatment; only one patient needed both radiotherapy and chemotherapy [8], while one needed chemotherapy only [11]. Additional treatment with GnRH-agonist was administered in one case [11], whereas surgery resolved precocious puberty in all the other cases. Gass et al. [10] reported a glioma of the quadrigeminal plate with symptoms of PP and hydrocephalus, successfully treated with endoscopic third ventriculostomy (ETV), given the infiltration of the dorsal midbrain (Table S1).

## 3. Discussion

GGs are rare primary central nervous system (CNS) tumors and represent around 5% of all pediatric CNS tumors, slightly more frequent in males [15,16] Within the pediatric population, the mean age of onset is between 10 and 11 years of age [16]. GGs are well-differentiated slow-growing tumors, composed of a mixed population of neoplastic glial and dysplastic neural cells [17,18]. They are usually located in the supratentorial compartment, mostly in the temporal lobes [14,15]. Nonetheless, they may arise around deep brain structures, such as brainstem or spinal cord [16]. The localization of GG in the posterior cranial fossa, as in our case, is atypical [19,20,21]. Symptoms of GGs generally depend on the localization: epileptic seizures, deficits due to a local mass effect and signs of increased intracranial pressure (ICP) are typically signs of supratentorial involvement, whereas infratentorial GGs may present with cerebellar signs, cranial nerve deficits or rarely increased ICP [16,21,22]. In our patient, CPP was the only clinical manifestation, whose gradual onset and absence of other signs determined a diagnostic delay, 1 year and a half after the appearance of pubic hair.

Several reports indicate that brain tumors are the most common neoplastic cause of CPP. Wendt et al. [12] collected records of all patients with a tumor associated with PP, treated at St. Jude Children’s Research Hospital, Memphis, TN, USA, from January 1975 through October 2011. Of 13,615 patients, 24 had PP and 12 were affected by brain tumors. Signs of PP were observed for 3 months or more before tumor diagnosis in 10 patients and emerged before tumor-related signs and symptoms. In our case, a multidisciplinary approach prompted by a pediatric endocrinology consult led to a timely diagnosis of the tumor after confirming CPP. However, signs of abnormal puberty were overlooked before hospital consultation, causing a 1.5-year delay in the evaluation of PP and, therefore, in brain tumor diagnosis.

Most brain tumors causing CPP are located in the sellar-suprasellar, hypothalamic and pineal regions [3,12]. In such cases CPP is believed to be caused by premature activation of the hypothalamic–pituitary–gonadal axis [3]. Intracranial tumors located elsewhere and presenting with hydrocephalus may exert a mass effect on the hypothalamus, which is thought to contribute to pubertal GnRH release and subsequent secretion of LH and FSH [3,9]. In our case, the GG was located in the posterior fossa but was not associated with elevated ICP. Five cases of CPP in children with posterior fossa tumors have been previously reported and, for those without hydrocephalus, endocrinological mechanisms leading to CPP are uncertain (Table S1). As assumed by Josan et al. [9], CPP might be caused by subclinical raised ICP impairing the inhibitory pathways to GnRH pulse generator.

Independently from histological diagnosis, surgical resection was the cornerstone of treatment in four out of five cases and gross total resection was a positive prognostic factor. As reported by Medina et al. [8], Josan et al. [9] and Gass et al. [10], the treatment of associated CPP was the surgery itself in three cases and no specific adjunctive treatment was required after tumor excision. In our case, the patient’s pubertal signs were long-standing and hormonal replacement therapy was necessary, with progressive clinical benefit.

## 4. Conclusions

CPP associated with posterior fossa tumors are rare entities. Here we describe a unique case of CPP in a patient with a posterior fossa GG. Our work highlights the importance of prompt specialist referral in all cases of PP, regardless of clinical neurological signs or symptoms, in order to detect possible tumor association and guide management by a multidisciplinary approach.

## Data Availability

Not applicable.

## Supplementary Materials

The following are available online at https://www.mdpi.com/article/10.3390/neurolint13040053/s1, Table S1: Posterior fossa tumor and central precocious puberty: reported cases in the literature.
