# Supplementary material for: A Rare Case of Posterior Fossa Tumor and Central Precocious Puberty: Case Presentation and Review of the Literature
Source: Neurol Int. 2021 Oct 20;13(4):535–40. doi: 10.3390/neurolint13040053 (PMC8544465; doi:10.3390/neurolint13040053)
Supplement: Supplementary file 1 [file neurolint-13-00053-s001.zip › neurolint-1422430-supplementary.pdf]

Table S1. Posterior fossa tumor and central precocious puberty: reported cases in the literature

Reference	No.	Sex And Age	Brain Tumor Histology	Location	Endocrinological Symptoms at Presentation	Hydrocephalus at Presentation	Endocrinological Therapy	Tumor Treatment
Our case	1	Male, 11	Ganglioglioma	Cerebellum	pubarche, incremented testicular volume,height > 95°, advanced bone age	no	GnRH agonist (triptorelina)	Surgery
Medina et al. [8]	2	Male, 7	Medulloblastoma	Cerebellum	pubarche, incremented testicular volume, acne, height and weight >95°	yes	none	Surgery, chemotherapy, radiotherapy
Josan et al. [9]	3	Female, 2	Pilocytic astrocytoma	Cerebellum	vaginal bleeding	no	none	Surgery
Gass et al. [10]	4	n.a.	Glioma	Tectal plate	n.a.	yes	none	ETV
Rossfeld et al. [11]	5	Male, 7	Medulloblastoma	Cerebellum	pubarche, axillary hair, body odor	n.a.	GnRH agonist (leuprolide)	Surgery, chemotherapy
Wendt et al. [12]	6	Female, 7	Pilocytic astrocytoma	Cerebellum	menarche, advanced bone age	n.a.	n.a.	Surgery

CPP = central precocious puberty; n. a. = not available; ETV= endoscopic third ventriculostomy
